# Raymond Edward Ryckman

**DOI:** 10.3201/eid2208.151678

**Published:** 2016-08

**Authors:** Rachel Curtis-Robles, Charles B. Beard

**Affiliations:** Texas A&M University, College Station, Texas, USA (R. Curtis-Robles);; Centers for Disease Control and Prevention, Fort Collins, Colorado, USA (C.B. Beard)

**Keywords:** Chagas disease, Triatoma, plague, bibliography, parasites, *Trypanosoma cruzi*

This is a photograph of Raymond Edward Ryckman, PhD, (Figure) a medical entomologist. His studies of triatomine bugs, *Trypanosoma cruzi*, and Chagas disease formed a rich library of information about vectors and hosts of *T. cruzi*, including the behavioral ecology of the vectors and the role of pack rats for sustaining *T. cruzi* in natural habitats.

**Figure:**
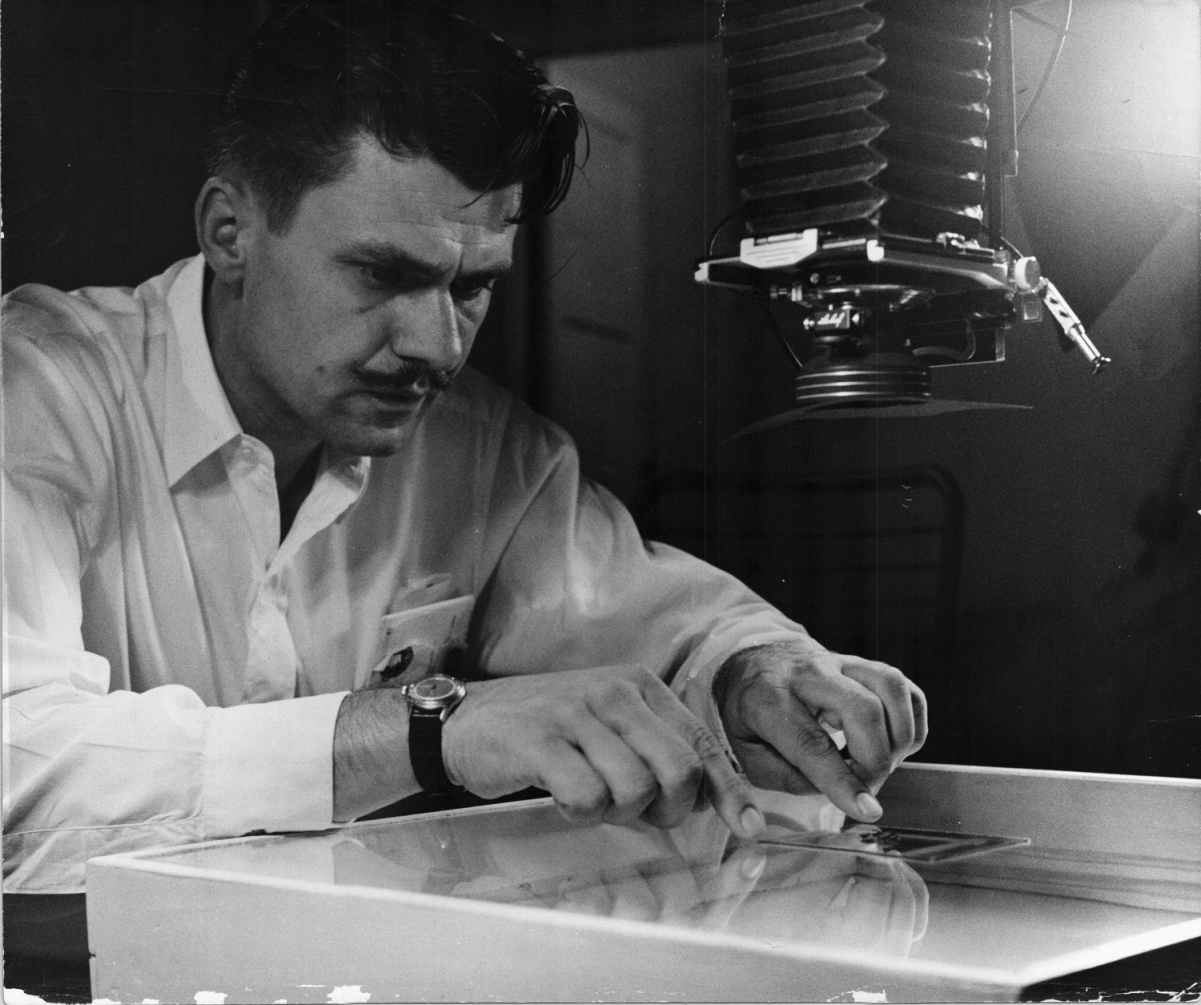
Raymond Edward Ryckman

Ryckman was born June 19, 1917, on a farm in southern Wisconsin, USA; fascinated at an early age by insects, he built an observation beehive and was eager to show the colony to visitors. He was drafted during World War II and served 4 years at the Presidio Army Base hospital in San Francisco, California, USA. After receiving a bachelor of science degree in zoology at the University of California, Berkeley, in 1950, Ryckman was recruited to teach at Loma Linda University in Loma Linda, California.

Soon after arriving at Loma Linda, he was approached by the Army Surgeon General’s office and asked to research the dynamics of plague transmission, with regard to the Army’s interest in troop health and safety in Southeast Asia. Ryckman used the natural plague system in southern California to study squirrel and flea population dynamics and potential insecticides. The innovative methods he used included electric-fence enclosures, ferrets trained to place devices within squirrel burrows, and crucible tongs used to gently handle unmanageable squirrels (*1*–*3*). His research played a major role in the understanding of the dynamics of plague transmission and control.

After the Army Surgeon General’s plague grant ended, Ryckman returned to the University of California, Berkeley, where he completed his master’s thesis on Cimicidae (bed bugs) and forged ahead with PhD studies under Dr. Robert Usinger. His research explored the systematics, hybridization, and reproduction of the triatomine *Triatoma protracta*—vector of *T. cruzi*, the agent of Chagas disease (*4*). He completed his PhD research while fulfilling teaching requirements at Loma Linda University. He educated hundreds of medical and graduate students at the School of Medicine at Loma Linda University until his retirement in 1987.

In addition to being a triatomine researcher, he was a naturalist eager to investigate organisms that captured his interest, generally incidental to studies of triatomines. During his career he published articles about cactiphilic flies (*5*) and lizard mites (*6*). After his retirement, he continued to write, co-authoring a book about Edmund C. Jaeger, a naturalist who studied the desert ecology of the southwestern United States (*7*).

Ryckman traveled throughout Central and South America, generally returning with field-collected triatomines to start new colonies. His family frequently traveled with him, and he published several articles with his sons (*8*–*10*). Ryckman credits his wife with careful and patient review of his manuscripts before submission. He authored or co-authored ≈115 publications, most of which were 1- or 2-author publications.

Later in Ryckman’s career, his focus turned to the publication of bibliographies. Before Internet and electronic searching were available, bibliographies were valuable sources of information for researchers, and their collation was a time-intensive, although perhaps underappreciated, achievement. In Ryckman’s words, “A bibliographic monograph is the summation of our historical, cultural, and scientific heritage in a given field of endeavor” (*11*). His career capstone was the publication of bibliographies “concerned with the world literature to the Triatominae and Triatominae-borne pathogens and clinical Chagas’ disease” (*12*). Careful curation was achieved with the help of assistants, requests via reprint request cards, use of a shopping cart to transport journals between the university library and copy shop, and use of a punch card system to organize articles. Compiled over 16 years and containing >23,000 publications, these bibliographies are a unique contribution to the field of Chagas disease research (*11*–*13*). The hard copy (print) collection resides at the Centers for Disease Control and Prevention in Atlanta, Georgia, USA.

In addition to a legacy of bibliographies and publications, Ryckman’s collection of >25,000 insects is available for study at the Bohart Museum of Entomology, University of California, Davis. This collection includes the triatomine specimens that resulted from Ryckman’s many colonies of triatomines and other insects.

In honor of his contributions to the study of Chagas disease vectors, Ryckman was honored with an eponym in 1972: *Triatoma ryckmani*, a rare species from Central America (*14*). As an authority on triatomine ecology and Chagas disease, a patient teacher and mentor, an international scholar, and a family man, Raymond E. Ryckman is an admirably well-rounded scientist.
